# Comparison of cervical versus thoracic spinal cord injury outcomes in pediatric trauma patients

**DOI:** 10.1007/s00383-024-05933-4

**Published:** 2025-02-26

**Authors:** Alice M. Martino, Areg Grigorian, Catherine M. Kuza, Sigrid Burruss, Lourdes Swentek, Yigit Guner, Laura F. Goodman, Jeffry Nahmias

**Affiliations:** 1 Department of Surgery, University of California Irvine Medical Center, 333 The City Blvd West, Suite 1600, Orange, CA 92868-3298 USA; 2 Division of Trauma, Burns, Critical Care and Acute Care Surgery, University of California Irvine Medical Center, Orange, CA USA; 3 Department of Anesthesiology and Critical Care Medicine, Harbor-UCLA Medical Center, Torrance, CA USA; 4 Department of Pediatric Surgery, Children’s Hospital of Orange County, Orange, CA USA

**Keywords:** Pediatric trauma, Spinal cord injury, Mortality, Substance use

## Abstract

**Purpose:**

To explore differences based on level of pediatric spinal cord injury (SCI), we compared cervical and thoracic SCI in pediatric trauma patients (PTPs), hypothesizing higher mortality and length of stay (LOS) for cervical SCI.

**Methods:**

The 2017–2021 Trauma Quality Improvement Program was queried for all PTPs ≤ 17 years-old with cervical or thoracic SCI. Bivariate analyses compared the two groups. The primary outcome was mortality and secondary outcomes included hospital LOS and injury severity scores (ISS). Logistic regression models were used to determine independent risk factors for death and prolonged ventilation.

**Results:**

Of 5280 PTPs, 2538 (65.9%) had cervical SCI and 1316 (34.1%) had thoracic SCI. Motor vehicle collisions were the most common cause of both cervical and thoracic SCI (37.8 and 41.9%). PTPs with thoracic SCI had higher rates of positive drug screen as compared to cervical SCI (39.2 vs 29.8%, *p* = 0.001). PTPs with thoracic SCI had higher median ISS (25 vs 16, *p* < 0.001), while cervical SCI had higher mortality (13 vs 6.1%, *p* < 0.001) but decreased hospital LOS (median 9 vs 5 days, *p* < 0.001. Cervical SCI were associated with a nearly fourfold increase in the risk of death (95% CI 2.750–5.799, *p* < 0.001) and a 1.6-fold increase in the risk of prolonged ventilator requirement (95% CI 1.228–2.068, *p* < 0.001).

**Conclusions:**

PTPs with cervical SCI have higher mortality while those with thoracic SCI have higher ISS and hospital LOS. Cervical SCI were associated with a fourfold higher risk of death. MVC was the most common cause of injury, and both groups had high rates of positive drug screens. Understanding differing outcomes may assist providers with prognostication and injury prevention.

**Supplementary Information:**

The online version contains supplementary material available at 10.1007/s00383-024-05933-4.

## Introduction

Spinal cord injury (SCI) in the pediatric population is uncommon, affecting only 1.5% of injured children [1, 2]. Due to embryologic and anatomical differences, children’s spines are more vulnerable to injury than those of adults. In children eight years and younger, the spine is hypermobile and due to ligamentous laxity, shallow facet joints, and incomplete ossification, the risk of ligamentous injury is higher than for adults [3, 4]. Young children are also at particular risk for SCI because their spines are able to stretch more than their spinal cord—one study found that while the spine of an infant can stretch up to five centimeters, the spinal cord ruptures after only 5–6 mm of traction [5]. Additionally, the proportionally larger heads of children shifts the fulcrum of injury upwards, leading to more frequent high cervical spine injuries compared to adults [3, 4].

Based on previous small studies, cervical SCI is more common than thoracic SCI, especially in children eight years and younger [2, 6]. The thoracic spine is the least commonly injured region in this age group [6]. However, data regarding injury patterns in pediatric SCI remain limited, as studies tend to broadly focus on spinal injury or are limited to a specific portion of the spinal column (e.g., cervical). One single center study of pediatric spine injuries found that location of injury correlated with distinct injury patterns: upper cervical spine injuries were associated with orthopedic injuries, lower cervical spine was associated with maxillofacial injury, and the lower thoracic spine was associated with gastrointestinal injury [7]. Furthermore, while data on adult spine injuries exist, demonstrating that higher spine injuries correlate with higher mortality and longer hospital stays, similar detailed data for the pediatric population are lacking [8, 9]. Given the paucity of data directly comparing cervical and thoracic SCI in the pediatric population and the potential for differences in outcomes, we sought to utilize a national database to describe cervical and thoracic SCI in children and compare outcomes. We hypothesized that cervical SCI would be associated with higher mortality and hospital length of stay (LOS), compared to thoracic SCI.

## Methods

This study involved a retrospective analysis of the American College of Surgeons Trauma Quality Improvement Program (TQIP) database. We queried the database for trauma patients ≤17 years of age admitted to participating trauma centers from 2017–2021 with a cervical or thoracic SCI, as identified by International Classifications of Diseases (ICD) version-10 diagnosis codes. Complete and incomplete lesions were identified by ICD-10 codes (see Supplemental Table 1). Patients with both a cervical and thoracic SCI, as well as those with lumbar SCIs, were excluded from analysis. This allowed us to compare patients with only cervical SCI to patients with only thoracic SCI. This study was deemed exempt by our Institutional Review Board and a waiver of consent granted, as the study utilized de-identified patient data from the TQIP database.

The primary outcome was mortality. Secondary outcomes included hospital LOS, intensive care unit (ICU) LOS, ventilator days, and major complications (stroke, cardiac arrest, unplanned intubation, pulmonary embolism (PE), deep vein thrombosis (DVT), catheter-associated urinary tract infection (CAUTI), central line associated blood stream infection (CLABSI), and surgical site infections). To account for survivor bias, hospital LOS was only analyzed among survivors. Additional data variables collected were age, sex, and comorbidities including attention deficit/hyperactivity disorder (ADHD), mental disorders (schizophrenia, bipolar disorder, major depressive disorder, social anxiety disorder, posttraumatic stress disorder, antisocial personality disorder), diabetes, pre-trauma steroid use, and drug and alcohol screen results. The injury profile of patients was characterized by mechanism of injury such as motor vehicle collisions (MVCs), falls, auto versus pedestrian, bicycle and motorcycle collisions, gunshot wounds and stab wounds, as well as concomitant injuries by organ system and injury severity score (ISS). Vital signs on arrival were also collected. For the purposes of this study, hypotension was defined as systolic blood pressure <90 mmHg, tachypnea was defined as a respiratory rate >22 breaths per minute, and tachycardia was defined as a heart rate of >120 beats per minute.

### Statistical analysis

Continuous variables were reported as medians with interquartile ranges and were analyzed using the Mann–Whitney U test. Categorical variables were reported as frequencies and analyzed using chi square test. A *p* value <0.05 was set as the threshold for statistical significance. A multivariable logistic regression model was utilized to identify independent risk factors for death and prolonged intubation. Prolonged intubation was defined as 4 days or longer. This model included variables that were chosen by co-author consensus after review of the literature [10–12] and that were deemed significant in the univariate analysis with a *p*-value of <0.20. The model controlled for age, injury severity, hypotension, tachycardia, smoking history, and penetrating trauma. All *p*-values were two sided, with a statistical significance level of <0.05. All analyses were performed with IBM SPSS Statistics for Windows (Version 29, IBM Corp., Armonk, NY).

## Results

### Demographics and injury profiles of children with cervical versus thoracic SCI

Of 3,854 pediatric trauma patients, 2,538 (65.9%) had cervical SCI and 1,316 (34.1%) had thoracic SCI. Compared to children with thoracic SCI, those with cervical SCI were slightly younger (median 14 vs 15 years, *p* < 0.001). Both groups were predominantly male (68.8 vs 68.1%, *p* = 0.66). Children with cervical SCI less commonly had mental disorders (3.7 vs 6.0%, *p* = 0.001), smoking (2.3 vs 4.6%, *p* < 0.001), and positive drug screens (29.8 vs 39.2%, *p* = 0.001, Table 1). 341 cervical SCI and 386 thoracic SCI were identified as complete, and 565 cervical SCI and 297 thoracic SCI were identified as incomplete (Supplemental Table 2).

**Table 1:** Demographics of pediatric trauma patients with cervical versus thoracic spinal cord injury (SCI)

Characteristic	Cervical SCI n = 2538 (65.9%)	Thoracic SCI n = 1316 (34.1%)	*p*-value
Age (years), median (IQR)	14 (6)	15 (5)	<0.001*
Male, n (%)	1741 (68.8%)	895 (68.1%)	0.669
Comorbidities, n (%)			
ADHD	158 (6.3%)	76 (5.9%)	0.608
Mental disorder	94 (3.7%)	78 (6.0%)	0.001*
Smoking	58 (2.3%)	59 (4.6%)	<0.001*
Congenital anomalies	66 (2.6%)	21 (1.6%)	0.050
Diabetes	9 (0.4%)	8 (0.6%)	0.255
Functionally dependent	12 (0.5%)	1 (0.1%)	0.045*
Steroid use	2 (0.1%)	2 (0.2%)	0.499
Alcohol screen positive	74 (7.8%)	60 (9.9%)	0.143
Drug screen positive	203 (29.8%)	176 (39.2%)	0.001*

*ADHD* attention deficit hyperactivity disorder

^*^ denotes statistical significance, *p* value <0.05

Cervical SCI was more often caused by falls (26.1 vs 23.8%, *p* < 0.001) and auto vs pedestrian (4.7 vs 2.8%, *p* = 0.005) and less commonly resulted from MVC (37.8 vs 41.9%, *p* = 0.014), gunshot wounds (6.6 vs 26.7%, *p* < 0.001), and motorcycle collisions (1.4 vs 2.7%, *p* = 0.004) compared to thoracic SCI. Patients with cervical SCI were less often hypotensive (11.1 vs 13.8%, *p* = 0.018), tachypneic (31.2 vs 39.3%, *p* < 0.001), and tachycardic (15.1 vs 21.8%, *p* < 0.001) on arrival compared to thoracic SCI. Patients with cervical SCI had a lower median ISS than those with thoracic SCI (16 vs 25, *p* < 0.001) (Table 2).

**Table 2:** Injury profile of pediatric trauma patients with cervical versus thoracic spinal cord injury (SCI)

Characteristic	Cervical SCI n = 2538 (65.9%)	Thoracic SCI n = 1316 (34.1%)	*p*-value
Mechanism, n (%)			
Motor vehicle collision	959 (37.8%)	551 (41.9%)	0.014*
Fall	662 (26.1%)	207 (23.8%)	<0.001*
Gunshot wound	167 (6.6%)	352 (26.7%)	<0.001*
Auto vs pedestrian	119 (4.7%)	37 (2.8%)	0.005
Bicycle collision	68 (2.7%)	35 (2.7%)	0.971
Motorcycle collision	36 (1.4%)	36 (2.7%)	0.004*
Stab wound	7 (0.3%)	5 (0.4%)	0.582
Initial vital signs, n (%)			
Hypotensive (SBP < 90 mmHg)	273 (11.1%)	177 (13.8%)	0.018*
Tachypneic (RR > 22)	753 (31.2%)	497 (39.3%)	<0.001*
Tachycardic (heart rate > 120)	375 (15.1%)	283 (21.8%)	<0.001*
ISS, median (IQR)	16 (20)	25 (16)	<0.001*
Concomitant Injuries, n (%)			
Brain	702 (27.7%)	215 (16.3%)	<0.001*
Cardiac	28 (1.1%)	27 (2.1%)	0.019*
Lung	614 (24.4%)	712 (54.1%)	<0.001*
Rib	212 (8.4%)	422 (32.1%)	<0.001*
Diaphragm	17 (0.7%)	44 (3.3%)	<0.001*
Liver	152 (6.0%)	136 (10.3%)	<0.001*
Spleen	118 (4.6%)	114 (8.7%)	<0.001*
Kidney	70 (2.8%)	92 (7.0%)	<0.001*
Pancreas	21 (0.8%)	19 (1.4%)	0.073
Esophagus	2 (0.1%)	13 (1.0%)	<0.001*
Stomach	5 (0.1%)	20 (1.5%)	<0.001*
Small intestine	63 (2.5%)	39 (3.0%)	0.377
Colon	42 (1.7%)	39 (3.0%)	0.007*
Rectum	5 (0.2%)	3 (0.2%)	0.841
Bladder	8 (0.3%)	7 (0.5%)	0.306
Upper extremity fracture	161 (6.3%)	124 (9.4%)	<0.001*
Lower extremity fracture	191 (7.5%)	106 (8.1%)	0.559

*SBP* systolic blood pressure

*RR* respiratory rate

*ISS* injury severity score

*IQR* interquartile range

^*^ denotes statistical significance, *p* value <0.05

Cervical SCI patients had higher rates of brain injury compared to thoracic SCI (27.7 vs 16.3%, *p* < 0.001), but lower rates of most other injuries including lung (24.4 vs 54.1%, *p* < 0.001), ribs (8.4 vs 32.1%, *p* < 0.001), and many abdominal organs (Table 2).

### Complications and outcomes of children with cervical versus thoracic SCI

Compared to thoracic SCI patients, cervical SCI patients had higher mortality (13.0 vs 6.1%, *p* < 0.001). However, cervical SCI patients had a shorter median hospital LOS (5 vs 9 days, *p* < 0.001). In terms of other outcomes, rates of DVT (1.1 vs 2.0%, *p* = 0.040) and PE (0.1 vs 0.2%, *p* = 0.021) were lower in patients with cervical SCI compared to those with thoracic SCI. Patients with cervical SCI also had lower rates of unplanned return to the operating room (1.1 vs 1.9%, *p* = 0.033) but increased ventilator days (6 vs 4, *p* < 0.001) (Table 3). After excluding patients who died during their hospitalization, cervical SCI continued to have lower rates of DVT (2.0 vs 1.2%, *p* = 0.049) and unplanned return to operating room (2.0 vs 1.2%, *p* = 0.048), while rates of PE were no longer significantly different. Ventilator days remained significantly higher in those with cervical SCI (9 vs 4, *p* < 0.001), and ICU LOS was significantly higher in those with cervical SCI (6 vs 5 days, *p* = 0.009) (Supplemental Table 3).

**Table 3:** Complications and outcomes of pediatric trauma patients with cervical versus thoracic spinal cord injury (SCI)

Characteristic	Cervical SCI n = 2538 (65.9%)	Thoracic SCI n = 1316 (34.1%)	*p*-value
Complications, n (%)			
Delirium	10 (1.8%)	8 (2.8%)	0.369
Stroke	7 (0.3%)	7 (0.5%)	0.211
Cardiac arrest	82 (3.2%)	29 (2.2%)	0.070
Unplanned intubation	55 (2.2%)	31 (2.4%)	0.711
Ventilator-associated pneumonia	59 (2.3%)	30 (2.3%)	0.932
Respiratory	23 (0.9%)	16 (1.2%)	0.364
Pulmonary embolism	3 (0.1%)	6 (0.2%)	0.040*
Kidney	10 (0.4%)	8 (0.6%)	0.357
Deep vein thrombosis	27 (1.1%)	26 (2.0%)	0.021*
CAUTI	24 (0.9%)	17 (1.3%)	0.322
Sepsis	11 (0.4%)	10 (0.8%)	0.192
Superficial surgical site infection	3 (0.1%)	3 (0.2%)	0.413
Deep surgical site infection	4 (0.2%)	4 (0.3%)	0.345
CLABSI	5 (0.2%)	2 (0.2%)	0.755
Unplanned return to operating room	25 (1.1%)	24 (1.9%)	0.033*
Any complication	328 (12.9%)	196 (14.9%)	0.091
Mortality in hospital, n (%)	329 (13.0%)	80 (6.1%)	<0.001*
Hospital LOS, median (IQR)	5 (12)	9 (13)	<0.001*
ICU LOS, median (IQR)	5 (10)	5 (7)	0.101
Ventilator Days, median (IQR)	6 (14)	4 (9)	<0.001*

*CAUTI* catheter associated urinary tract infection

*CLABSI* central line associated bloodstream infection

*LOS* length of stay

*ICU* intensive care unit

*IQR* interquartile range

^*^ denotes statistical significance, *p* value <0.05

### Logistic regression models for risk of death and prolonged intubation

When controlling for ISS, age, hypotension, tachycardia, smoking history, and penetrating mechanism, cervical SCI was associated with a nearly fourfold increased risk of death (95% CI 2.750–5.799, *p* < 0.001). ISS (OR 1.057, 95% CI 1.049–1.064, *p* < 0.001), penetrating mechanism (OR 3.117, 95% CI 2.104–4.618, *p* < 0.001), and hypotension (OR 9.660, 95% CI 7.101–13.141, *p* < 0.001) were also independent risk factors for death (Table 4A). Cervical SCI was also associated with a 1.6-fold higher risk for prolonged intubation (95% CI 1.228–2.068, *p* < 0.001) (Table 4B).

**Table 4A:** Logistic regression model predicting death in children with SCI

	Odds Ratio	95% Confidence interval		*p*-value
Cervical SCI	3.994	2.750	5.799	<0.001*
ISS	1.057	1.049	1.064	<0.001*
Age (years)	0.919	0.893	0.945	<0.001*
Hypotension (SBP < 90)	9.660	7.101	13.141	<0.001*
Tachycardia (HR > 120)	1.215	0.870	1.696	0.252
Smoking history	0.541	0.176	1.668	0.285
Penetrating trauma	3.117	2.104	4.618	<0.001*

**Table 4B:** Logistic regression model predicting prolonged intubation in children with SCI

	Odds Ratio	95% Confidence interval		*p*-value
Cervical SCI	1.594	1.228	2.068	<0.001*
ISS	1.014	1.007	1.022	<0.001*
Age (years)	0.988	0.965	1.011	0.306
Hypotension (SBP < 90)	0.617	0.462	0.825	0.001*
Tachycardia (HR > 120)	0.774	0.595	1.008	0.057
Smoking history	0.810	0.412	1.592	0.541
Penetrating trauma	1.027	0.752	1.402	0.867

*SCI* spinal cord injury

*ISS* injury severity score

*SBP* systolic blood pressure

*HR* heart rate

Prolonged intubation defined as 4 or more days

^*^ denotes statistical significance, *p* value < 0.05

## Discussion

SCI in pediatric patients, although rare, results in significant lifelong disabilities and medical costs estimated at 4.5 million dollars over a lifetime [6]. This retrospective national database review demonstrated that falls more commonly resulted in cervical SCI than thoracic SCI, while MVCs, gunshot wounds, auto vs pedestrian, and motorcycle collisions more often led to thoracic SCI. Furthermore, substance and alcohol use were more common in children with thoracic SCI. Finally, this study demonstrated higher mortality and ventilator days for children with cervical SCI, however children with thoracic SCI had higher overall injury burden (e.g., ISS) and hospital LOS.

Substance use disorder has a known correlation with traumatic injury in both adults [13–17] and children [18, 19]. The current analysis found that over 30% of children with SCI had a positive drug screen and nearly 10% tested positive for alcohol. This is corroborated by several studies that have demonstrated substance use disorder as a risk factor for SCI in adults [20–22], as well as one study of both adult and pediatric patients with brain and spine injuries resulting from ATV crashes, which found nearly 50% of cases involved alcohol intoxication [23]. Our study also demonstrates that thoracic SCI was more likely to be associated with both alcohol and substance use than cervical SCI. Therefore, while pediatric patients are not just small adults, they similarly have injuries linked to substance and alcohol use and thus would benefit from increased primary prevention efforts.

Notably MVC was the most common mechanism for both cervical and thoracic SCI, followed by falls, which is consistent with prior studies in children [1, 24, 25]. Interestingly, MVCs resulted in more thoracic SCI than cervical SCI. Existing pediatric literature has predominantly focused on cervical spine injuries resulting from MVC. One 10 year review of the National Pediatric Trauma Registry found cervical spine injury was rare (1.5% of the study population), yet cervical SCI was even more rare, comprising only 35% of those with spinal injury [1]. Importantly, 73% of their cohort was injured in a MVC, and 61% of these children were unrestrained at the time of their injury [1]. Similarly, MVC remains a significant mechanism for thoracolumbar spine injuries in children as well, comprising 55% of these injuries, followed by falls (23%) [26]. Given that MVCs remain the biggest contributors to pediatric SCI of both the cervical and thoracic spine, it is clear that injury prevention efforts, including correct use of car seats/seat belts, are still needed. In addition, the automobile industry and federal agencies responsible for car seat regulations should leverage this data to try and further optimize these restraint devices to minimize SCIs in children.

Higher levels of spinal cord injury generally imply increased level of motor deficit and potential for increased complications [27]. Thus, not surprisingly, outcomes also differed between cervical and thoracic SCI. Cervical SCI were associated with a more than doubled mortality rate compared to thoracic SCI (13 vs 6%) but had a shorter median hospital LOS by four days. Our mortality rate for cervical SCI is comparable to previous work by Brown et al. [28] (18.5%) for cervical spine injuries at their center. Interestingly, children with cervical SCI in our national analysis had a lower median ISS [16] compared to those in the Brown study (ISS = 25) and children in our cohort with thoracic SCI (ISS = 25). The lower ISS in our cervical SCI cohort may be due to survivor bias, as children who died shortly after arrival, likely due to severe injury or high cervical SCI (which is often fatal [2]) may not have undergone complete trauma imaging evaluations. Additionally, the higher LOS and survivorship may help explain why thoracic SCI patients had increased complications such as sepsis and venous thromboembolism. However, after excluding patients who died during their hospital stay, only DVT rates remained elevated for patients with thoracic SCI. More granular scene and prospective hospital data are needed to definitively determine the reason for the differences in mortality and complications.

Our study has limitations inherent to its nature as a retrospective database analysis, such as missing data, coding errors, and misclassification. Children with severe injuries that did not survive to hospitalization were not included in the TQIP database. In addition, the TQIP database lacks granular data related to the severity of the SCI including the American Spinal Injury Association (ASIA) classification let alone even a distinction for complete versus incomplete SCI. Also, it lacks pertinent variables known to effect outcomes in SCI patients such as timing of surgical decompression and degree of neurologic recovery. Finally, the TQIP database lacks long-term post-discharge data, such as post hospitalization development of scoliosis, pressure ulcers, and patient centric outcomes including quality of life measurements. As SCI is a lifelong condition, more long-term data would be useful to paint a complete picture of the differences between cervical and thoracic SCI. Despite these limitations, this study’s large number of patients and centers adds to the generalizability of the findings.

## Conclusions

In conclusion, this four-year retrospective analysis of pediatric trauma patients in the TQIP database demonstrated differences in mechanism of injury and injury pattern between cervical and thoracic SCI, although MVC was the most common mechanism for both injuries. Cervical SCI was associated with higher mortality whereas thoracic SCI patients had higher ISS, complications, and hospital LOS. These findings highlight the urgent need for enhanced injury prevention strategies, especially encouraging safe driving practices among parents, as MVCs continue to be a major cause of SCI in children. Future research is needed to improve injury prevention, especially related to MVCs, and evaluate differences in long-term outcomes, with the goal of improving the treatment and quality of life for children with SCI.

## Supplementary Information

Below is the link to the electronic supplementary material.

Supplementary file1 (DOCX 23 KB)

Supplementary file2 (JPG 268 KB)

## Data Availability

The data that support the findings of this study are not publicly available but may be obtained by request from the American College of Surgeons Committee on Trauma.
