# Supplementary material for: Comparison of cervical versus thoracic spinal cord injury outcomes in pediatric trauma patients
Source: Pediatr Surg Int. 2025 Feb 26;41(1):86. doi: 10.1007/s00383-024-05933-4 (PMC11865126; doi:10.1007/s00383-024-05933-4)
Supplement: Supplementary file 1 — Supplementary file1 (DOCX 23 KB) [file 383_2024_5933_MOESM1_ESM.docx]

Supplemental Table 1: ICD-10 codes for complete and incomplete cervical and thoracic SCI

| **Complete cervical SCI** | **Complete thoracic SCI** | **Incomplete cervical SCI** | | **incomplete thoracic SCI** |
| --- | --- | --- | --- | --- |
| S14.11 | S24.11 | S14.12 | S14.14 | S24.13 |
| S14.111 | S24.111 | S14.121 | S14.141 | S24.131 |
| S14.111A | S24.111A | S14.121A | S14.141A | S24.131A |
| S14.111D | S24.111D | S14.121D | S14.141D | S24.131D |
| S14.111S | S24.111S | S14.121S | S14.141S | S24.131S |
| S14.112 | S24.112 | S14.122 | S14.142 | S24.132 |
| S14.112A | S24.112A | S14.122A | S14.142A | S24.132A |
| S14.112D | S24.112D | S14.122D | S14.142D | S24.132D |
| S14.112S | S24.112S | S14.122S | S14.142S | S24.132S |
| S14.113 | S24.113 | S14.123 | S14.143 | S24.133 |
| S14.113A | S24.113A | S14.123A | S14.143A | S24.133A |
| S14.113D | S24.113D | S14.123D | S14.143D | S24.133D |
| S14.113S | S24.113S | S14.123S | S14.143S | S24.133S |
| S14.114 | S24.114 | S14.124 | S14.144 | S24.134 |
| S14.114A | S24.114A | S14.124A | S14.144A | S24.134A |
| S14.114D | S24.114D | S14.124D | S14.144D | S24.134D |
| S14.114S | S24.114S | S14.124S | S14.144S | S24.134S |
| S14.115 | S24.119 | S14.125 | S14.145 | S24.139 |
| S14.115A | S24.119A | S14.125A | S14.145A | S24.139A |
| S14.115D | S24.119D | S14.125D | S14.145D | S24.139D |
| S14.115S | S24.119S | S14.125S | S14.145S | S24.139S |
| S14.116 |  | S14.126 | S14.146 | S24.14 |
| S14.116A |  | S14.126A | S14.146A | S24.141 |
| S14.116D |  | S14.126D | S14.146D | S24.141A |
| S14.116S |  | S14.126S | S14.146S | S24.141D |
| S14.117 |  | S14.127 | S14.147 | S24.141S |
| S14.117A |  | S14.127A | S14.147A | S24.142 |
| S14.117D |  | S14.127D | S14.147D | S24.142A |
| S14.117S |  | S14.127S | S14.147S | S24.142D |
| S14.118 |  | S14.128 | S14.148 | S24.142S |
| S14.118A |  | S14.128A | S14.148A | S24.143 |
| S14.118D |  | S14.128D | S14.148D | S24.143A |
| S14.118S |  | S14.128S | S14.148S | S24.143D |
| S14.119 |  | S14.129 | S14.149 | S24.143S |
| S14.119A |  | S14.129A | S14.149A | S24.144 |
| S14.119D |  | S14.129D | S14.149D | S24.144A |
| S14.119S |  | S14.129S | S14.149S | S24.144D |
|  |  | S14.13 | S14.15 | S24.144S |
|  |  | S14.131 | S14.151 | S24.149 |
|  |  | S14.131A | S14.151A | S24.149A |
|  |  | S14.131D | S14.151D | S24.149D |
|  |  | S14.131S | S14.151S | S24.149S |
|  |  | S14.132 | S14.152 | S24.15 |
|  |  | S14.132A | S14.152A | S24.151 |
|  |  | S14.132D | S14.152D | S24.151A |
|  |  | S14.132S | S14.152S | S24.151D |
|  |  | S14.133 | S14.153 | S24.151S |
|  |  | S14.133A | S14.153A | S24.152 |
|  |  | S14.133D | S14.153D | S24.152A |
|  |  | S14.133S | S14.153S | S24.152D |
|  |  | S14.134 | S14.154 | S24.152S |
|  |  | S14.134A | S14.154A | S24.153 |
|  |  | S14.134D | S14.154D | S24.153A |
|  |  | S14.134S | S14.154S | S24.153D |
|  |  | S14.135 | S14.155 | S24.153S |
|  |  | S14.135A | S14.155A | S24.154 |
|  |  | S14.135D | S14.155D | S24.154A |
|  |  | S14.135S | S14.155S | S24.154D |
|  |  | S14.136 | S14.156 | S24.154S |
|  |  | S14.136A | S14.156A | S24.159 |
|  |  | S14.136D | S14.156D | S24.159A |
|  |  | S14.136S | S14.156S | S24.159D |
|  |  | S14.137 | S14.157 | S24.159S |
|  |  | S14.137A | S14.157A |  |
|  |  | S14.137D | S14.157D |  |
|  |  | S14.137S | S14.157S |  |
|  |  | S14.138 | S14.158 |  |
|  |  | S14.138A | S14.158A |  |
|  |  | S14.138D | S14.158D |  |
|  |  | S14.138S | S14.158S |  |
|  |  | S14.139 | S14.159 |  |
|  |  | S14.139A | S14.159A |  |
|  |  | S14.139D | S14.159D |  |
|  |  | S14.139S | S14.159S |  |

SCI: spinal cord injury

Supplemental Table 2: Frequencies of complete and incomplete cervical and thoracic SCI

|  | **Cervical** | **Thoracic** |
| --- | --- | --- |
| **Complete lesion** | 341 | 386 |
| **Incomplete lesion** | 565 | 297 |

SCI: spinal cord injury

Supplemental Table 3: Complications and outcomes of pediatric trauma patients with cervical versus thoracic spinal cord injury (SCI) who survived to hospital discharge

| **Characteristic** | **Cervical SCI**  **n = 2203 (64.1%)** | **Thoracic SCI**  **n = 1234 (35.9%)** | **p-value** |
| --- | --- | --- | --- |
| Complications, n (%) |  |  |  |
| Delirium | 9 (1.9%) | 8 (3.0%) | 0.352 |
| Stroke | 5 (0.2%) | 5 (0.4%) | 0.352 |
| Cardiac arrest | 36 (1.6%) | 13 (1.1%) | 0.168 |
| Unplanned intubation | 54 (2.5%) | 29 (2.3%) | 0.852 |
| Ventilator-associated pneumonia | 50 (2.4%) | 29 (2.3%) | 0.874 |
| Respiratory | 16 (0.7%) | 15 (1.2%) | 0.146 |
| Pulmonary embolism | 3 (0.1%) | 6 (0.5%) | 0.054 |
| Kidney | 7 (0.3%) | 6 (0.5%) | 0.440 |
| Deep vein thrombosis | 26 (1.2%) | 25 (2.0%) | 0.049* |
| CAUTI | 21 (1.0%) | 16 (1.3%) | 0.349 |
| Sepsis | 10 (0.5%) | 8 (0.6%) | 0.448 |
| Superficial surgical site infection | 3 (0.1%) | 3 (0.2%) | 0.471 |
| Deep surgical site infection | 4 (0.2%) | 4 (0.3%) | 0.406 |
| CLABSI | 4 (0.2%) | 2 (0.2%) | 0.896 |
| Unplanned return to operating room | 24 (1.2%) | 24 (2.0%) | 0.048* |
| Any complication | 263 (11.9%) | 176 (14.2%) | 0.049* |
| Hospital LOS, median (IQR) | 5 (12) | 9 (13) | <0.001* |
| ICU LOS, median (IQR) | 6 (11) | 5 (7) | 0.009* |
| Ventilator Days, median (IQR) | 9 (20) | 4 (9) | < 0.001* |

CAUTI: catheter associated urinary tract infection

CLABSI: central line associated bloodstream infection

LOS: length of stay

ICU: intensive care unit

IQR: interquartile range

* denotes statistical significance, p value < 0.05
