# Supplementary figures and images for: Comparison of cervical versus thoracic spinal cord injury outcomes in pediatric trauma patients
Source: Pediatr Surg Int. 2025 Feb 26;41(1):86. doi: 10.1007/s00383-024-05933-4 (PMC11865126; doi:10.1007/s00383-024-05933-4)

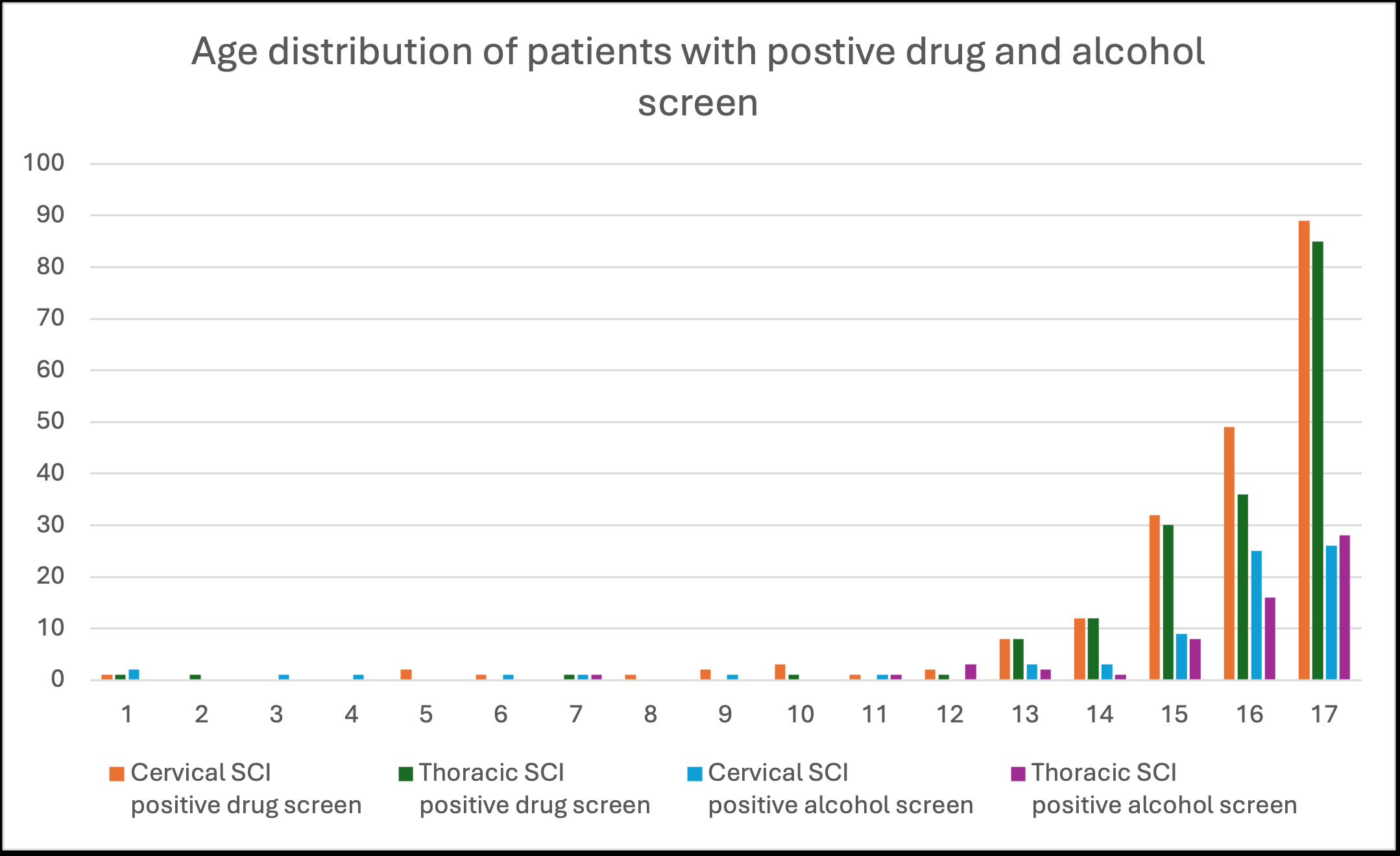

Supplement: Supplementary file 2 — Supplementary file2 (JPG 268 KB) [file 383_2024_5933_MOESM2_ESM.jpg]
